# Antifungal Agents: Design, Synthesis, Antifungal Activity and Molecular Docking of Phloroglucinol Derivatives

**DOI:** 10.3390/molecules23123116

**Published:** 2018-11-28

**Authors:** Xingxing Teng, Yuanyuan Wang, Jinhua Gu, Peiqi Shi, Zhibin Shen, Lianbao Ye

**Affiliations:** 1 School of Pharmacy, Guangdong Pharmaceutical University, Guangzhou 510006, China; tengxingxing563@126.com (X.T.); 15768985885@163.com (Y.W.); huaqianphar@126.com (J.G.); spq15989276019@163.com (P.S.); 2 School of Traditional Chinese Medicine, Guangdong Pharmaceutical University, Guangzhou 510006, China; szb8113@126.com; 3 Key Laboratory of New Drug Discovery and Evaluation of Ordinary Universities of Guangdong Province, Guangzhou Key Laboratory of Construction and Application of New Drug Screening Model Systems, Guangzhou key Laboratory of Construction and Application of New Drug Screening Model Systems and Guangdong Province Engineering Technology Center for Molecular Probes & Biomedical Imaging, Guangdong Pharmaceutical University, Guangzhou 510006, China

**Keywords:** phloroglucinol derivatives, antifungal activity, molecular docking, allylamine, squalene epoxidase

## Abstract

Pseudoaspidinol is a phloroglucinol derivative with Antifungal activity and is a major active component of *Dryopteris fragrans*. In our previous work, we studied the total synthesis of pseudoaspidinol belonging to a phloroglucinol derivative and investigated its antifungal activity as well as its intermediates. However, the results showed these compounds have low antifungal activity. In this study, in order to increase antifungal activities of phloroglucinol derivatives, we introduced antifungal pharmacophore allylamine into the methylphloroglucinol. Meanwhile, we remained C1–C4 acyl group in C-6 position of methylphloroglucinol using pseudoaspidinol as the lead compound to obtain novel phloroglucinol derivatives, synthesized 17 compounds, and evaluated antifungal activities on *Trichophyton rubrum* and *Trichophyton mentagrophytes* in vitro. Molecular docking verified their ability to combine the protein binding site. The results indicated that most of the compounds had strong antifungal activity, in which compound **17** were found to be the most active on *Trichophyton rubrum* with Minimum Inhibitory Concentration (MIC) of 3.05 μg/mL and of *Trichophyton mentagrophytes* with MIC of 5.13 μg/mL. Docking results showed that compounds had a nice combination with the protein binding site. These researches could lay the foundation for developing antifungal agents of clinical value.

## 1. Introduction

Fungi are widely distributed in nature and frequently present as pathogens in the animal and plant kingdoms [1]. In recent decades, despite progress in antifungal therapy, infectious diseases caused by a variety of clinically significant species of fungi remain a major global health concern, due to the development of antifungal drug resistance [2,3]. However, resistance to several antifungal agents among a variety of clinically significant species of fungi is becoming an increasingly major global problem and is reaching an alarming level on a global scale [4]. Hence, searching for efficient, nontoxic or low toxic chemotherapeutic agents with potent broad-spectrum antifungal activity is an important goal in new drug research [5,6]. Currently, the main targets of the antifungal agents are the cytochrome P450 sterol 14a-demethylase (CYP51), squalene epoxidase (SE) and β-1,3-glucan synthase, in which azole and triazole drugs are CYP51 inhibitors widely used as fungal antibiotics and antimycobacterial activity, spinosins are β-1,3-glucan synthase inhibitors and allylamine agents act on squalene epoxidase (SE) [7,8,9,10].

**Figure 1:**
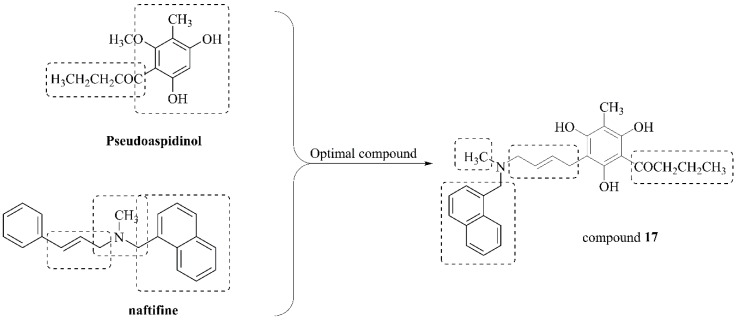
Design of compounds.

*Dryopteris fragrans* is mainly used as a folk medicine at present. It has been found that the main active compounds from *Dryopteris fragrans* have a significant effect on a variety of skin diseases caused by fungi [11,12]. Our group isolated a variety of phloroglucinols from the plant and activity experiment showed that the phloroglucinols had strong antifungal activity such as the minimal inhibition concentration (MIC) values activity of aspidin BB against some clinical *S. aureus* was ranged from 15.63 mg/mL to 62.5 mg/mL [13,14,15,16]. In addition, in our current research, phlorglucinols, especially 2-methyl-6-acyl phloroglucinol derivatives. Pseudoaspidinol belongs to phlorglucinols and shows good antifungal activity and its key intermediate methyl phlorglucinol can be used to generate diverse compounds. In our previous study, a preliminary structure-activity study found that the hydroxyl group of methyl phloroglucinol and the butyryl group at the C-4 position are the active groups of the compound, and C-4 or C-6 is the active site. Introduction of different groups at this position may alter its properties and biological activity. In order to increase antifungal activity of phloroglucinol derivatives, in this study, we introduced antifungal pharmacophore allylamine into the methyl phloroglucinol and remained C1–C4 acyl group in C-6 position using pseudoaspidinol as lead compound to obtain novel phloroglucinol derivatives 4–17 (Figure 1). 

We found in the literature that the main antifungal target of allylamine antifungal drugs is squalene oxidase, especially the representative drug naftifine [17]. It is particularly noteworthy that naftifine is the first marketed drug for allylamine antifungal drugs and is not replaceable in antifungal action. Unfortunately, the widespread use of allylamine has led to the development of severe drug resistance, which has significantly reduced their efficacy [18]. These compounds were synthesized and evaluated for their antifungal activity against *Trichophyton rubrum* and *Trichophyton mentagrophytes* in vitro. Molecular docking simulations were conducted to confirm the mechanism of action between the compound and squalene epoxidase (SE).

## 2. Results and Discussions

### 2.1. Chemistry

**Scheme 1:**
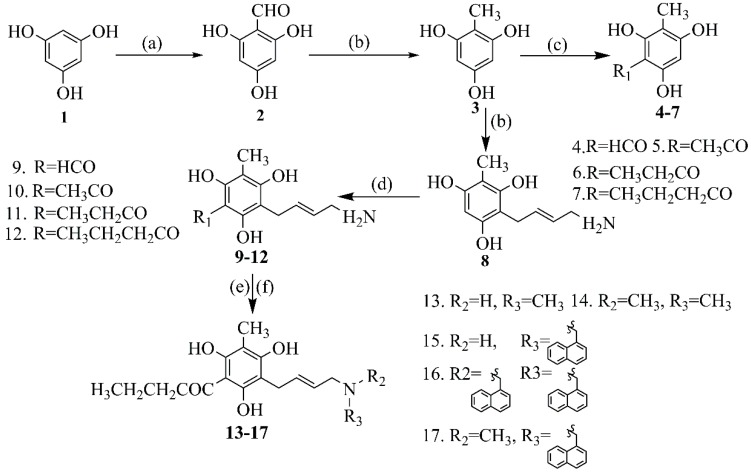
Synthesis of phloroglucinol derivatives. (a) POCl_3_, DMF, EtoAc. (b) NaBH_3_CN, THF, PH 4. (c) AlCl_3_, CS_2_, reflux. (d) AlCl_3_, CH_2_Cl_2_, rt. (e) CH_3_I, K_2_CO_3_, DMF, reflux. (f) PEG-400, K_2_CO_3_, KI, EtOH, reflux.

The synthetic route of compounds was demonstrated in Scheme 1. Methyl phloroglucinol (**3**) was synthesized using 1,3,5-hydroxybenzene (**1**) as raw material through Vilsmeier–Haack reaction and reduction reaction. The formyl chloride, acetyl chloride, propionyl chloride and butyryl chloride are introduced into the C-4 position of methyl phloroglucinol under the catalysis of aluminum trichloride and nitrobenzene to obtain the compound **4**–**7**. In addition, we are at normal temperature conditions. The 4-chloro-2-butenamine hydrochloride is introduced into the benzene ring of the compound **3** to obtain the compound **8**. Compound **8** reacted with the above four acid chloride to make compound **9**–**12** by Friedel–Crafts reaction using AlCl_3_ as catalyst under mild reaction conditions. On the one hand, compound **12** is achieved in DMF by adding potassium carbonate and methyl iodide to give compounds **13** and **14** under reflux. On the other hand, the methylnaphthalene group of compounds **15**–**16** were obtained by reacting a compound **12**, 1-Chloromethyl naphthalene in the presence of K_2_CO_3_ and potassium iodide in ethanol. Finally, 1-chloromethylnaphthalene is introduced into a compound **12** to give the final product compound **17**.

In our previous studies, the reduction of aldehyde groups on the benzene ring to methyl groups mostly uses NaBH_3_CN as a catalyst and tetrahydrofuran as a solvent, but this reduction reaction is sensitive to pH, and the reaction at room temperature requires at least 10 h [1]. In the subsequent synthesis optimization, we used zinc powder as a catalyst, diethyl ether and ethyl acetate as a common solvent. This improvement makes the whole reaction not need to adjust pH, and the normal temperature reaction can be completely finished within one hour—even the yield increased from 60% to 90%. It is well known that Friedel–Crafts reaction is part of the most important reactions. In the past, when butyryl group was introduced into methyl phloroglucinol, if only AlCl_3_ and a single solvent carbon disulfide were unreactive, the addition of nitrobenzene had a momentous influence on the reaction. In this study, there are hydroxyl groups on both sides of the C-4 position of methyl phloroglucinol. This electron donating group will offset the passivation of the acyl ring to the benzene ring. The second acyl group is submitted at the C-6 position in the reaction, so the reaction yield is low. It is worth mentioning that after the introduction of an acyl group on the benzene ring of the compound **4**–**7**, the acyl group as the electron withdrawing group was completely inert to Friedel-Crafting. Subsequently, we introduce 4-chloro-2-butenamine hydrochloride into the C-6 position of compound **2** to obtain compound **8**, then carry out acylation reaction to obtain compound **9**–**12**. Surprisingly, the synthesis of compound **9**–**12** did not require the addition of nitrobenzene, and the solvent was changed from carbon disulfide to dichloromethane. This reaction stilled be and the yield was partially improved. This change is more in line with the contemporary concept of green chemistry. Compound **12** has a lone pair of electrons on the nitrogen atom, so it is easy to methylate, then the ratio of methyl iodide and DMF is changed to obtain compounds **13** and **14**. After exploring various methods, we add PEG-400 and potassium iodide, enhancing the activity of 1-chloromethylnaphthalene and introducing a methylnaphthalene into the nitrogen atom of compound **12** under mild normal temperature conditions to obtain a secondary amine compound **15** and a tertiary amine compound **16**. In the final synthesis of compound **17**, methylnaphthalene is more likely to be included onto compound **13** than when a methyl group is introduced on compound **15**. Since the introduction of a methyl group by a secondary amine usually requires refluxed reaction, this causes instability of the naphthalene and excessive impurities.

### 2.2. Evaluation of Antifungal Activity

**Figure 2:**
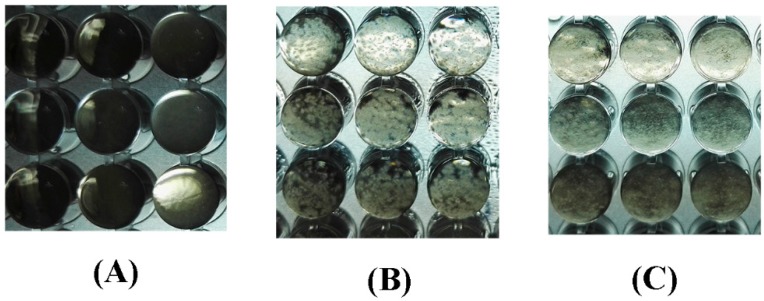
(**A**) Complete antifungal effect. (**B**) Growth effect of fungal strains: *Trichophyton rubrun*. (**C**) Growth effect of fungal strains: *Trichophyton mentagrophytes*.

The antifungal results of all synthetic compounds, pseudoaspidinol and Naftifine were shown in Table 1. The reading of the minimum inhibitory concentration results complied with by observing the growth effect of the two fungal strains on the 96-well plate (seen in Figure 2). Pseudoaspidinol exhibited strong activity against *T. mentagrophyte* and *T. rubrum*, with IC_50_ values of 21.06 μg/mL and 19.89 μg/mL, respectively. Compound **3** exhibited poor activity against *T. mentagrophytes* and *T. rubrum*. Subsequently, the activity of the compound **4**–**7** showed that only the compound **6** and the compound **7** had an antifungal effect. The antifungal activity of compound **6** was 62.5 μg/mL, while the IC_50_ of compound **7** against *T. rubrum* was 39.89 μg/mL. Activity of against *T. mentagrophytes* was similar. Disappointingly, Compound **8** was lower active against both fungi. The activity of Compound **12** was not optimized compared to the activity of Compound **7**, but the activity of Compound **12** was decreased by nearly 1/3 compared with Compound **6**, and the activity against both fungi was 39.24 μg/mL and 39.17 μg/mL, respectively. Surprisingly, the activity of Compound **9** was practically four times lower than that of Compound **4**. Compounds **15** and **17** showed the best activity against *Trichophyton rubrum* and *T. mentagrophyte*, and the antifungal effect was much higher than that of the lead compound pseudomicinol.

**Figure 3:**
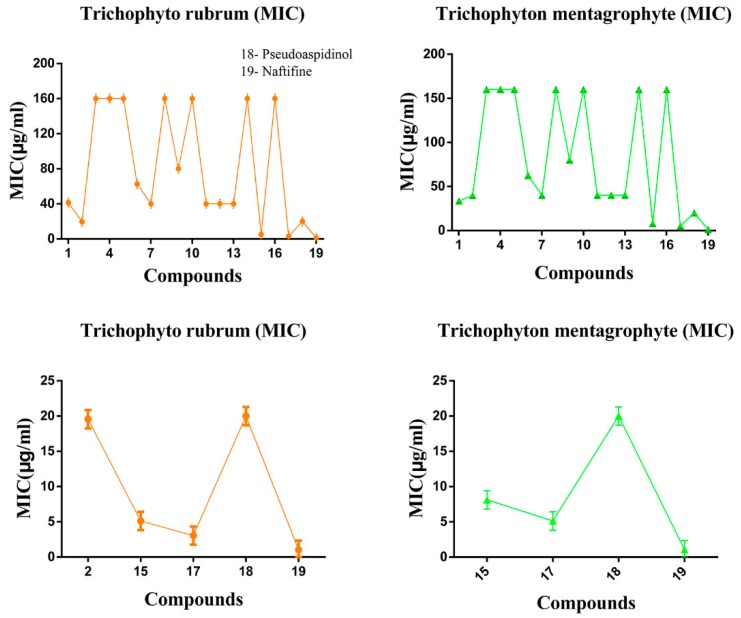
Activities of all compounds against *Trichophyton rubrun* and *Trichophyton mentagrophytes*. (**A**) The antifungal results of all compounds against *Trichophyton rubrun*. (**B**) The antifungal results of all compounds against *Trichophyton mentagrophytes*. (**C**) The strong antifungal results of compounds against *Trichophyton rubrun*. (**D**) The strong antifungal results of compounds against *Trichophyton mentagrophytes*.

The results showed that the butyryl group at the C-4 position of methyl phloroglucinol may be a pharmacophore of pseudomicinol. Subsequently, it was found that most of the antifungal activity results of the compound **9**–**12** after the para-introduction of the acyl group after the introduction of the allylamine group at the C-6 position of the methyl phloroglucinol skeleton was optimized (Figure 3A,B). This clearly indicates that the allylamine group may not be suitable as a single antifungal group, but it has some antifungal optimization effects. In addition, the naphthylmethyl group of N in allylamine can improve the antifungal activity. Obviously, structurally modified compounds **15** and **17** exhibited excellent activity against *Trichophyton rubrum* and *T. mentagrophyte*, compared to pseudomicinol (Figure 3C,D). From the optimal compound **17**, it can be assumed that the antifungal key pharmacophore of the allylamine antifungal agent such as Naftifine is methylnaphthalene. Further, research work on structure-activity relationship is currently under investigation and will be reported in due course.

### 2.3. Docking Studies

**Figure 4:**
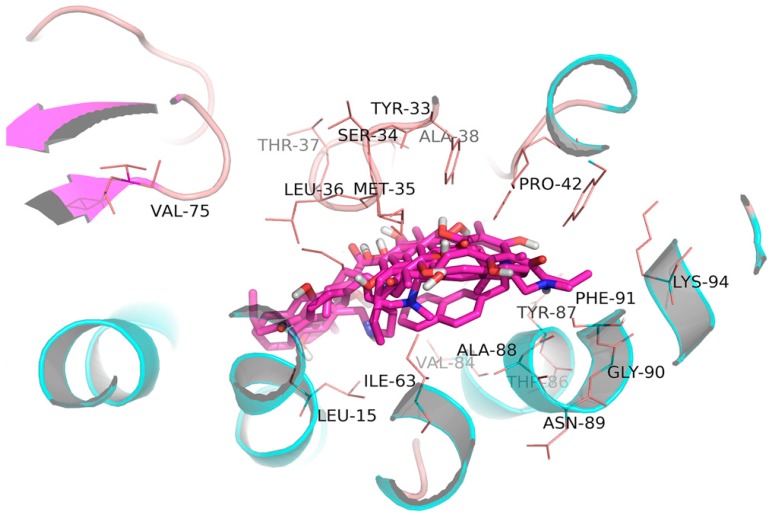
Binding modes of compounds **13**–**17** with 2AIB. The structural framework of compounds was marked with amaranth color. Protein skeleton of 2AIB was marked with green color. The atoms involved in the path are identified as colored stick. (Oxygen (orange), nitrogen (blue), hydrogen in hydroxyl (white)).

**Figure 5:**
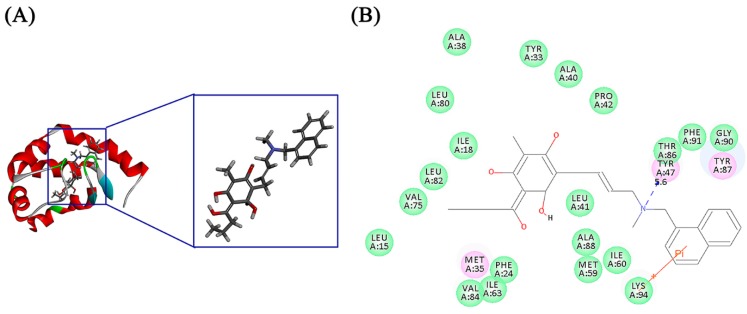
(**A**) Optimal binding modes of compound **17** in squalene epoxidase (PDB ID 2AIB); (**B**) 2D interaction plot of 2AIB and compound **17**. The structural framework of compound **17** was marked with green color. Protein skeleton of 2AIB was marked with red color. The atoms involved in the path are identified as colored stick. (Oxygen (red), nitrogen (blue), hydrogen (white)).

**Figure 6:**
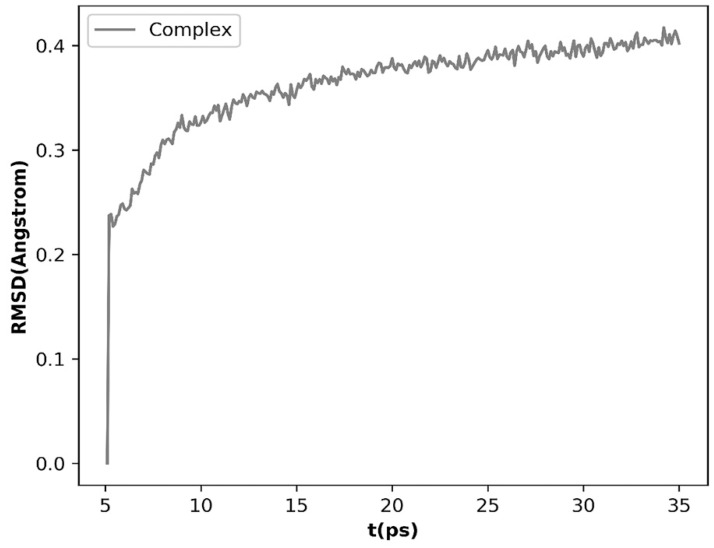
Plots of RMSD for all of the backbone atoms (Å) vs. simulation time (ps) for 2AIB in complex with compound **17**.

Currently, the main targets of the antifungal agents are the cytochrome P450 sterol 14a-demethylase (CYP51), squalene epoxidase (SE) and β-1,3-glucansynthase. The mechanism of molecular docking was targeted at the positive drug, Naftifine. Docking experiments select SE (PDB ID: 2AIB) to perform molecular docking since allylamine antifungal agents mainly acts on SE [7,8,9,10]. The docking score of compounds is shown in Table 2. The results show compounds **4**–**13** had higher scores than pseudoaspidinol in accord with the results of antifungal activities and compound **8**–**17** demonstrate better score and antifungal activities, and the 3D interactions of compounds **9**–**13** with 2AIB are shown in Figure 4. As can be seen from the diagram, compounds formed the hydrogen bonding interactions of allylamine NH with TYR 47 and phenolic hydroxyl with MET 35 and TYR 33, hydrophobic interaction of allyl with LEU 41, MET 59 and ALA 88, and Pi-Pi interaction of naphthalene ring with LYS 94. Compound **17** shows the best antifungal activity owing to Pi-Pi interaction of a naphthalene ring and hydrophobic interactions of N–CH_3_ with ILE 60 (seen in Figure 5). In order to understand the binding mode in a dynamic environment, molecular dynamics simulation was executed for the compound **17** with the crystal structure of 2AIB. Compound **17** exhibited a stable binding conformation throughout the MD run as confirmed through the ligand Root Mean Squared Deviation (RMSD), and the ligand tends to stable during 15–35 ps (RMSD < 0.4 Å) (Figure 6).

## 3. Materials and Methods 

### 3.1. Synthesis of Compounds

The solvents and reagents used throughout this study were purchased from commercial vendors and were dried and purified by conventional methods prior to use. Nuclear magnetic resonance (NMR) spectra was recorded on a Bruker AC-400P spectrometer (Bruker, Karlsruhe, Germany) with TMS as the internal standard and was analyzed by MestReNova (Mestrelab Research, Santiago de Compostela, Coruña, Spain) [19]. Infrared spectra were recorded using potassium bromide disks on a Hitachi 270-50 spectrophotometer (Hitachi Limited, Tokyo, Japan) and ESI mass spectra were made on an API-3000 LC-MS spectrometer (Applied Biosystems, Shanghai, China). Thin-Layer Chromatography (TLC) analysis was performed on silica gel 60 F254 silica plates (Merck KGaA, Darmstadt, Germany). ^1^H-NMR, ^13^C-NMR and MS of compounds **2**–**17** were found in Supplementary Materials.

*Synthesis of***3**. To the solution of 1-formyl-2,4,6-trihydroxy benzene (**2**) (1.54 g, 10 mmol) in tetrahydrofuran (20 mL) we added methyl orange (1–2 drops) followed by the addition of sodium cyanoborohydride (1.90 g, 30 mmol). The pH of the reaction mixture was maintained at 4.0, throughout the reaction, by addition of 1 N HCl. The reaction mixture was stirred at room temperature for 12 h prior to extraction with ethyl acetate (30 mL × 4). The ethyl acetate layer was washed with brine solution (30 mL × 3) and finally dried over Na_2_SO_4_. Solvent was evaporated to get the crude product, which was purified by column chromatography with hexane/EtOAc (50:50 *v*/*v*). 

*Synthesis of***7**. 2,4,6-trihydroxytoluene (2 g, 14.28 mmol) was placed in a 250 mL three-necked flask, carbon disulfide (10 mL) and anhydrous aluminum trichloride powder (5.7 g, 42.84 mmol) were added, and nitrobenzene (6 mL) was added with stirring, and heated to reflux. After 0.5 h, then butyryl chloride (1.68 g, 15.7 mmol) was slowly added dropwise, and the mixture was refluxed for 1.5 h, cooled to room temperature, and poured into a mixture of ice water (10 mL of concentrated hydrochloric acid, 70 g of ice water) with stirring, and distilled for 30 min. The mixture was filtered while hot, and the yellow needle-like crystals were precipitated in the filtrate. After sufficient cooling, the mixture was filtered, and the remaining oil was repeatedly subjected to hot extraction several times. The combined crystals were dried to obtain 2-methyl-6-butyrylphloroglucol.

*Synthesis of***8**. At room temperature, 2-methylbenzene-1,3,5-triol (421.04 mg, 3 mmol) and anhydrous aluminum trichloride powder (799.98 mg, 6 mmol) were slowly added to a solution of methylene chloride (20 mL), stirred evenly and refluxed for 1 h. Simultaneously, 4-chloro-2-butenamine hydrochloride (468.41 mg, 3.31 mmol) was dissolved in a 10% (by mass) aqueous sodium hydroxide solution(10 mL), stirred for 10 min to neutralize the hydrochloride, extracted three times(15 mL) with methylene chloride and dried over anhydrous sodium sulfate. The neutralized solution of 4-chloro-2-butenamine in methylene chloride was slowly added to a flask containing 2-methylbenzene-1,3,5-triol at 0 °C. At room temperature, the reaction was stirred until complete. The solvent was recovered, dissolved in deionized water, extracted with ethyl acetate five times (20 mL), dried over anhydrous sodium sulfate and the ethyl acetate removed under reduced pressure to obtain the crude product as a yellow solid. This crude product was isolated by silica gel column chromatography (hexane/EtOAc 1:1 *v*/*v*) to obtain compound **8**.

*Synthesis of***11**. A mixture of 8 (627.62 mg, 3 mmol) and aluminum chloride (1.201 g, 9 mmol) in 20 mL of methylene chloride was added to propionyl chloride (305 mg, 3.3 mmol). The reaction mixture was stirred at 42 °C and monitored by TLC. Once the reaction was complete (5 h), the mixture was poured onto ice water (70.00 g) and hydrochloric acid (2.00 g) was added. The solvent was recovered, dissolved in deionized water, extracted with ethyl acetate five times (20 mL), dried over anhydrous sodium sulfate and the ethyl acetate removed under reduced pressure to obtain the crude product. The residue was subsequently purified by silica gel column chromatography (hexane/EtOAc 3:2 *v*/*v*) to give a pure product.

*Synthesis of***13**. The intermediate of compound **12** (558.30 mg, 2 mmol) was then dissolved in dry DMF (20 mL), followed by the addition of K_2_CO_3_ (2.66 g, 12 mmol) and Me_2_SO_4_ (2.51 g, 12 mmol). The reaction mixture was refluxed for 4 h, after which the mixture was filtered to obtain compound **13**.

*Synthesis of***15**. PEG-400 (5 d) and potassium carbonate were added to a mixture of compound **12** (1.40 g, 5 mmol) in EtOH (15 mL). The solution of 1-Chloromethyl naphthalene (1.77 g, 10 mmol) and potassium iodide (1.60 g, 10 mmol) in EtOH (5 mL) was then added dropwise. The reaction was stirred at room temperature for 36 h and neutralized with a saturated solution of NaHCO_3_. Following extraction with dichloromethane, the organic layer was washed with brine, dried over anhydrous sodium sulfate, concentrated, and purified by column chromatography (methyl alcohol/dichloromethane, 1:20 *v*/*v*, *R*_f_ = 0.57) to obtain the intermediary of compound **15**.

*2,4,6-Trihydroxybenzaldehyde* (**2**). Yield: 82%. Yellow power. m.p.: 195.4–198.3 °C. ^1^H-NMR (400 MHz, DMSO): δ(ppm) 11.46 (s, 2H), 10.66 (s, 1H), 9.93 (s, 1H), 5.79 (s, 2H), 2.50 (dt, *J* = 3.5, 1.7 Hz, 1H). ^13^C-NMR (101 MHz, DMSO): δ(ppm) 192.4 (s), 167.5 (s), 165.7 (s), 159.3 (s), 102.9 (s), 94.9 (s), 87.9 (s). EI-MS: 153.36 [M − H]^−^. Anal. calcd for C_7_H_6_O_4_ (154.03): C, (54.34) 54.55; H, (3.91) 3.92; O, (41.75) 41.52.

*2-Methylbenzene-1,3,5-triol* (**3**). Yield: 78%. Yellow power. m.p.: 213.4–216.5 °C. ^1^H-NMR (400 MHz, DMSO): δ(ppm) 8.79 (s, 2H), 8.66 (s, 1H), 5.77 (s, 2H), 2.50 (s, 3H). ^13^C-NMR (101 MHz, DMSO): δ(ppm) 166.4 (s), 153.5 (d, *J* = 7.8 Hz), 103.1 (s), 94.0 (s), 86.3 (s), 8.2 (s). EI-MS: 139.52 [M − H]^−^. Anal. calcd for C_7_H_8_O_3_ (140.05): C, 60.00 (59.97); H, 5.75 (5.73); O, 34.25 (34.30).

*2,4,6-Trihydroxy-3-methylbenzaldehyde* (**4**). Yield: 58%. White solid. m.p.: 223.7–224.5 °C. ^1^H-NMR (400 MHz, DMSO): δ(ppm) 8.79 (s, 2H), 8.66 (s, 1H), 5.77 (s, 2H), 2.50 (s, 3H). ^13^C-NMR (101 MHz, DMSO): δ(ppm) 192.3 (s), 166.0 (s), 165.2 (s), 164.9 (s), 102.5 (s), 101.9 (s), 94.6 (s), 8.4 (s). EI-MS: 169.52 [M + H]^+^. Anal. calcd for C_8_H_8_O_4_ (168.04): C, 57.14 (57.08); H, 4.80 (4.83); O, 38.06 (38.09).

*1-(2,4,6-Trihydroxy-3-methylphenyl)ethan-1-one* (**5**). Yield: 76%. Yellow power. m.p.: 206.9–208.1 °C. ^1^H-NMR (400 MHz, DMSO): δ(ppm) 10.50 (s, 1H), 10.25 (s, 1H), 6.00 (s, 1H), 2.53 (s, 3H), 1.82 (s, 3H). ^13^C-NMR (101 MHz, DMSO): δ(ppm) 205.0 (s), 164.4 (d, *J* = 2.9 Hz), 163.1 (s), 104.0 (s), 102.8 (s), 94.3 (s), 28.6 (s), 7.8 (s). EI-MS: 183.90 [M + H]^+^. Anal. calcd for C_9_H_10_O_4_ (182.06): C, 59.34 (59.23); H, 5.53 (5.59); O, 35.13 (35.18).

*1-(2,4,6-Trihydroxy-3-methylphenyl)propan-1-one* (**6**). ^1^H-NMR (400 MHz, DMSO): δ(ppm) 10.48 (s, 1H), 10.20 (s, 1H), 6.00 (s, 1H), 2.99 (q, *J* = 7.2 Hz, 2H), 1.82 (s, 3H), 1.04 (t, *J* = 7.2 Hz, 3H). ^13^C-NMR (101 MHz, DMSO): δ(ppm) 206.1 (s), 163.7 (s), 162.7 (s), 160.2 (s), 103.8 (s), 101.8 (s), 94.4 (s), 36.8 (s), 9.3 (s), 7.7 (s). EI-MS: 197.93 [M + H]^+^. Anal. calcd for C_10_H_12_O_4_ (196.07): C, 61.22 (61.31); H, 6.17 (5.20); O, 32.62 (33.58).

*1-(2,4,6-Trihydroxy-3-methylphenyl)butan-1-one* (**7**). Yield: 68%. Yellow power. m.p.: 162.0–164.2 °C. ^1^H-NMR (400 MHz, DMSO): δ(ppm) 14.01 (s, 1H), 10.52 (s, 1H), 10.26 (s, 1H), 6.01 (s, 1H), 2.97 (t, *J* = 7.3 Hz, 2H), 1.84 (s, 3H), 1.60 (dd, *J* = 14.7, 7.3 Hz, 2H), 0.91 (t, *J* = 7.4 Hz, 3H). ^13^C-NMR (101 MHz, DMSO): δ(ppm) 205.1 (s), 163.4 (s), 162.3 (s), 159.7 (s), 103.5 (s), 101.4 (s), 94.0 (s), 45.1 (s), 17.8 (s), 13.9 (s), 7.3 (s). EI-MS: 211.53 [M + H]^+^. Anal. calcd for C_11_H_14_O_4_ (210.09): C, 62.85 (62.79); H, 6.71 (6.83); O, 30.44 (30.38).

*(E)-2-(4-Aminobut-2-en-1-yl)-4- methylbenzene-1,3,5-triol* (**8**). Yield: 95%. ^1^H-NMR (400 MHz, CDCl3): δ(ppm) 7.01 (s, 1H), 6.48 (s, 1H), 6.33–6.28 (m, 1H), 4.12 (dd, *J* = 14.3, 7.1 Hz, 1H), 2.24 (d, *J* = 34.0 Hz, 2H), 2.09 (d, *J* = 38.0 Hz, 2H), 1.57 (s, 3H). ^13^C-NMR (101 MHz, DMSO): δ(ppm) 156.5 (s), 155.6 (s), 153.2 (s), 129.6 (s), 123.0 (s), 107.2 (s), 101.7 (s), 94.5 (s), 46.6 (s), 25.4 (s), 8.4 (s). EI-MS: 209.56 [M − H]^−^. Anal. calcd for C_11_H_15_NO_3_ (209.11): C, 63.14 (63.12); H, 7.23 (7.24); N, 6.69 (6.71); O, 22.94 (22.93).

*(E)-3-(4-Aminobut-2-en-1-yl)-2,4,6-trihydroxy-5-methylbenzaldehyde* (**9**). Yield: 53%. ^1^H-NMR (400 MHz, DMSO): δ(ppm) 9.15 (s, 1H), 8.53 (s, 1H), 8.12 (s, 1H), 6.22 (dt, *J* = 24.2, 10.0, 1.5 Hz, 1H), 5.71 (dt, *J* = 24.1, 9.9, 1.6 Hz, 1H), 4.78 (s, 1H), 3.39–3.24 (m, 4H), 2.16 (s, 3H), 1.46 (s, 2H). ^13^C-NMR (101 MHz, DMSO): δ(ppm) 193.2 (s), 161.9 (s),156.8 (s), 155.9 (s), 129.6 (s), 123.0 (s), 106.4 (s), 101.2 (s), 94.5 (s), 46.6 (s), 24.8 (s), 8.4 (s). EI-MS: 236.21 [M − H]^−^. Anal. calcd for C_12_H_15_NO_4_ (237.10): C, 60.75 (60.73); H, 6.37 (6.38); N, 5.90 (5.89); O, 26.97 (27.00). 

*(E)-1-(3-(4-Aminobut-2-en-1-yl)-2,4,6-trihydroxy-5-methylphenyl)ethan-1-one* (**10**). Yield: 58%. ^1^H-NMR (400 MHz, DMSO): δ(ppm) 9.36 (s, 1H), 7.02 (s, 1H), 6.55 (s, 1H),5.65 (dt, *J* = 10Hz, 1H), 5.42 (dt, *J* = 9 Hz, 1H), 3.35 (d, *J* = 3Hz, 2H), 3.23 (d, *J* = 3 Hz, 2H), 2.32 (d, *J* = 4Hz, 6H), 1.26 (s, 2H). ^13^C-NMR (101 MHz, DMSO): δ(ppm) 205.7 (s), 162.1 (s), 161.6 (s), 161.2 (s), 129.6 (s), 123.0 (s), 107.0 (s), 106.6 (s), 104.5 (s), 46.6 (s), 28.6 (s), 24.8 (s), 8.1 (s). EI-MS: 251.73 [M + H]^+^. Anal. calcd for C_13_H_17_NO_4_ (251.12): C, 62.14 (61.13); H, 6.82 (6.83); N, 5.57 (5.56); O, 25.47 (27.48).

*(E)-1-(3-(4-Aminobut-2-en-1-yl)-2,4,6-trihydroxy-5-methylphenyl)propan-1-one* (**11**). Yield: 76%. Yellow power. m.p.: 155.6–157.4 °C. ^1^H-NMR (400 MHz, DMSO): δ(ppm) 8.65 (s, H), 8.53 (s, 1H), 5.83 (dt, *J* = 20.9, 12.3 Hz,1H), 5.57 (dt, *J* = 20.9, 9.1 Hz,1H), 4.25 (s, 1H), 3.64 (d, 2H), 3.62 (d, 2H), 2.64 (q, *J* = 8.4 Hz, 2H), 2.50 (s, 3H), 1.31 (t, *J* =8.5 Hz, 3H), 1.79 (s, 3H). ^13^C-NMR (101 MHz, DMSO): δ(ppm) 208.4 (s), 162.8 (s), 161.4 (d, *J* = 11.0 Hz), 129.6 (s), 123.0 (s), 106.6 (s), 106.1 (s), 102.8 (s), 46.6 (s), 35.5 (s), 24.8 (s), 8.4 (s), 8.1 (s). EI-MS: 265.55 [M + H]^+^. Anal. calcd for C_14_H_19_NO_4_ (265.13): C, 63.38 (63.39); H, 7.22 (7.23); N, 5.28 (5.27); O, 24.12 (24.11).

*(E)-1-(3-(4-Aminobut-2-en-1-yl)-2,4,6-trihydroxy-5-methylphenyl)butan-1-one* (**12**). Yield: 82%. White power. m.p.: 98.5–102.3 °C. ^1^H-NMR (400 MHz, DMSO): δ(ppm) 9.86 (s, 1H), 6.14 (dt, *J* = 24.3, 12.4 Hz, 1H), 5.71 (dt, *J* = 21.4, 11.4 Hz, 1H), 4.84 (s, 1H), 4.04 (s, 1H), 3.31 (m, *J* = 12.6, 4.1 Hz, 4H), 2.96 (t, *J* = 9.2 Hz, 2H), 2.16 (s, 3H), 1.60 (m, 2H), 1.43 (d, *J* = 65.2 Hz, 2H), 0.94 (t, *J* = 13.1 Hz, 3H). ^13^C-NMR (101 MHz, DMSO): δ(ppm) 205.5 (s), 163.8 (s), 162.7 (s), 160.1 (s), 129.6 (s), 123.0 (s), 104.0 (s), 101.8 (s), 94.4 (s), 45.5 (s), 41.8 (s), 24.8 (s), 18.3 (s), 14.3 (s), 7.7 (s). EI-MS: 279.57 [M + H]^+^. Anal. calcd for C_15_H_21_NO_4_ (279.15): C, 64.50 (64.49); H, 7.58 (7.59); N, 5.01 (4.99); O, 22.91 (22.93).

*(E)-1-(2,4,6-Trihydroxy-3-methyl-5-(4-(methylamino)but-2-en-1-yl)phenyl)butan-1-one* (**13**). Yield: 49%. ^1^H-NMR (400 MHz, DMSO): δ(ppm) 9.55 (s, 1H), 6.02 (dt, *J* = 11.2, 9.8 Hz, 1H), 5.71 (dt, *J* = 11.2, 9.84 Hz, 1H), 5.41 (s, 1H), 4.08 (s, 1H), 3.97 (s, 3H), 3.41–3.31 (m, 4H), 2.96 (t, *J* = 8.5 Hz, 2H), 2.16 (s, 3H), 1.67–1.47 (m, 2H), 1.34 (s, 1H), 0.94 (t, *J* = 10.48 Hz, 3H). ^13^C-NMR (101 MHz, DMSO): δ(ppm) 207.8 (s), 163.1 (s), 161.6 (d, *J* = 1.6 Hz), 127.3 (s), 124.6 (s), 106.7 (s), 106.5 (s), 103.4 (s), 55.4 (s), 41.8 (s), 36.2 (s), 24.8 (s), 19.6 (s), 13.5 (s), 8.1 (s). EI-MS: 293.81 [M + H]^+^. Anal. calcd for C_16_H_23_NO_4_ (293.16): C, 65.51 (65.50); H, 7.90 (7.91); N, 4.77 (4.76); O, 21.81 (21.83).

*(E)-1-(3-[4-(Dimethylamino)but-2-en-1-yl]-2,4,6-trihydroxy-5-methylphenyl)butan-1-one* (**14**). Yield: 51%. ^1^H-NMR (400 MHz, DMSO): δ(ppm) 7.75 (s, H), 6.04 (dt, *J* = 21.2, 11.3 Hz, 1H), 5.67 (dt, *J* = 18.2, 12.0, 9.04 Hz, 1H), 5.55 (s, 1H), 4.80 (s, 1H), 3.33 (dd, *J* = 9.8, 1.4 Hz, 2H), 3.08–2.90 (m, 4H), 2.75 (s, 6H), 2.16 (s, 3H), 1.68–1.45 (m, 2H), 0.94 (t, *J* = 10.5 Hz, 3H). ^13^C-NMR (101 MHz, DMSO): δ(ppm) 208.4 (s), 161.9 (d, *J* = 1.6 Hz), 159.3 (s), 157.7 (s), 131.7 (s), 126.8 (s), 111.8 (s), 106.5 (s), 106.5 (s), 49.1 (s), 46.3 (s), 45.4 (s), 25.4 (s), 17.1 (s), 13.8 (s), 7.4 (s). EI-MS: 307.57 [M + H]^+^. Anal. calcd for C_17_H_25_NO_4_ (307.18): C, 66.43 (66.42); H, 8.20 (8.21); N, 4.56 (4.58); O, 20.82 (20.79). 

*(E)-1-(2,4,6-Trihydroxy-3-methyl-5-(4-((naphthalen-2-ylmethyl)amino)but-2-en-1-yl)phenyl)butan-1-one* (**15**). Yield: 32%. ^1^H-NMR (400 MHz, DMSO): δ(ppm) 9.46 (s, 1H), 8.05–7.91 (m, 2H), 7.69 (dt, *J* = 11.8, 2.3 Hz, 1H), 7.56 (dt, *J* = 12.0, 2.4 Hz, 1H), 7.29 (dd, *J* = 18, 14.48, 7.28 Hz, 2H), 6.98 (dd, *J* = 12.0, 2.5 Hz, 1H), 6.22 (dt, *J* = 22.8, 9.9, 1.4 Hz, 1H), 5.78 (dt, *J* = 24.2, 10.0, 1.6 Hz, 1H), 5.01 (s, 1H), 4.20 (s, 2H), 3.66 (s, 1H), 3.49 (dd, *J* = 9.8, 1.6 Hz, 1H), 3.40 (dd, *J* = 9.84, 1.6 Hz, 1H), 3.33 (dd, *J* = 9.84, 1.4 Hz, 2H), 2.96 (t, *J* = 12.8 Hz, 2H), 2.16 (s, 4H), 1.69–1.45 (m, 2H), 0.94 (t, *J* = 10.6 Hz, 3H). ^13^C-NMR (101 MHz, DMSO): δ(ppm) 161.8 (s), 159.1 (s), 157.7 (s), 133.9 (s), 133.7 (s), 131.1 (s), 129.9 (s), 129.5 (s), 129.1 (s), 128.4 (s), 127.1 (d, *J* = 7.6 Hz), 125.9 (s), 124.3 (s), 111.0 (s), 106.8 (s), 106.1 (d, *J* = 34.1 Hz), 51.2 (s), 49.1 (s), 44.9 (s), 25.4 (s), 17.5 (s), 13.7 (s), 7.8 (s). EI-MS: 419.53 [M − H]^−^. Anal. calcd for C_26_H_29_NO_4_ (419.21): C, 74.44 (74.42); H, 6.97 (6.98); N, 3.34 (3.35); O, 15.25 (15.25).

*(E)-1-(3-(4-(bis(Naphthalen-2-ylmethyl)amino)but-2-en-1-yl)-2,4,6-trihydroxy-5-methylphenyl)butan-1-one* (**16**). Yield: 40%. ^1^H-NMR (400 MHz, DMSO): δ(ppm) 8.25–7.81 (m, 4H), 7.68 (dt, *J* = 12.0, 2.5 Hz, 2H), 7.55 (dt, *J* = 11.9, 2.4 Hz, 2H), 7.28 (dt, *J* = 17.9, 14.3, 7.2 Hz, 4H), 7.18 (s, 1H), 6.97 (dd, *J* = 12.0, 2.3 Hz, 2H), 6.92 (s, 1H), 6.25 (dt, *J* = 24.2, 9.9, 1.6 Hz, 1H), 5.72 (dt, *J* = 22.9, 9.8, 1.4 Hz, 1H), 4.68 (s, 1H), 4.09 (s, 4H), 3.32 (dd, *J* = 9.9, 1.5 Hz, 2H), 3.11–2.88 (m, 4H), 2.15 (s, 3H), 1.71–1.46 (m, 2H), 0.94 (t, *J* = 10.5 Hz, 3H). ^13^C-NMR (101 MHz, DMSO): δ(ppm) 208.8 (s), 161.6 (s), 159.2 (s), 157.5 (s), 138.0 (s), 137.2 (s), 133.4 (s), 132.1 (s), 131.9 (s), 130.1 (d, *J* = 26.4 Hz), 130.2–130.7 (m), 128.1 (d, *J* = 37.0 Hz), 128.2 (d, *J* = 13.1 Hz), 127.3 (d, *J* = 8.6 Hz), 126.4 (d, *J* = 29.7 Hz), 126.2 (d, *J* = 8.3 Hz), 126.1 (s), 125.7 (s), 124.4 (d, *J* = 22.4 Hz), 124.2 (s), 111.5 (s),106.5 (s), 106.3 (s), 60.2 (s), 53.4 (s), 49.5 (s), 46.5 (s), 25.7 (s), 17.6 (s), 13.8 (s), 7.5 (s).EI-MS: 559.53 [M − H]^−^. Anal. calcd for C_37_H_37_NO_4_ (559.27): C, 79.40 (79.39); H, 6.66 (6.68); N, 2.50 (2.49); O, 11.43 (11.44).

*(E)-1-(2,4,6-Trihydroxy-3-methyl-5-(4-(methyl(naphthalen-2-ylmethyl)aminbut-2-en-1-yl)phenyl)butan-1-one* (**17**). Yield: 75%. ^1^H-NMR (400 MHz, DMSO): δ(ppm) 8.47 (s, 1H), 8.05–7.89 (m, 2H), 7.69 (dt, *J* = 11.8, 2.3 Hz, 1H), 7.56 (dt, *J* = 11.9, 2.4 Hz, 1H), 7.28 (dd, *J* = 17.9, 14.3, 7.2 Hz, 2H), 6.98 (dd, *J* = 11.2, 2.4 Hz, 1H), 5.92 (dt, *J* = 21.8, 9.4, 1.3 Hz, 1H), 5.71 (dt, *J* = 24.2, 9.8, 1.3 Hz, 1H), 4.82 (s, 1H), 4.09 (s, 2H), 3.51 (s, 1H), 3.32 (dd, *J* = 9.7, 1.4Hz, 2H), 3.13–2.83 (m, 4H), 2.26 (s, 3H), 2.16 (s, 3H), 1.70–1.44 (m, 2H), 0.94 (t, *J* = 10.6 Hz, 3H). ^13^C-NMR (101 MHz, DMSO): δ(ppm) 207.0 (s), 162.5 (s), 162.0 (s), 136.5 (s), 136.3 (s), 133.4 (s), 132.5 (s), 131.7–131.0 (m), 127.6–129.2 (m), 128.5 (s), 126.9 (s), 106.7 (s), 106.1 (s), 103.4 (s), 60.3 (s), 58.7 (s), 47.8 (s), 47.5 (s), 24.9 (s), 19.7 (s), 13.6 (s), 8.1 (s). EI-MS: 433.30 [M − H]^−^. Anal. calcd for C_27_H_31_NO_4_ (433.23): C, 74.80 (74.79); H, 7.21 (7.19); N, 3.23 (3.22); O, 14.76 (14.80).

### 3.2. Evaluation of Antifungal Activity 

The antifungal activity of the compounds was evaluated against two standard human pathogenic fungal strains, *Trichophyton rubrum* (CMCC (F) T1d) and *Trichophyton mentagrophytes* (CMCC (F) T5c), purchased from the Chinese Academy of Medical Sciences (Hospital for Pathology and Dermatolog, Science and Peking Union Medical College). A solution of Naftifine (1.0 mg/mL) was used as a positive control, while pseudo mallow phenol (1.0 mg/mL) was used as a comparison. The minimum inhibitory concentration (MIC) of the compound was determined by a micro-broth dilution method according to the method defined by the National Clinical Laboratory Standards Committee (NCCLS National Clinical Laboratory Standards Committee 2002) [20]. For the assay, the MIC was determined in accordance with Clinical and Laboratory Standards Institute (CLSI) method M38-A2, in which the test compound monomer stock solution (32 mg/mL) and the positive drug naftifine were diluted 100-fold with RPMI-1640 liquid medium, and the concentration of the solvent DMSO after dilution (*v*/*v*) less than 1% to reduce the interference of the solvent on the experimental results. Then, the diluted compound monomer stock solution was subjected to lateral dilution in RPMI-1640 liquid medium. A blank control and growth control were placed in sterile 96-well plates. Finally, the 96 well plate mixed with the bacterial liquid and the drug solution was incubated at 35 °C for 4 days at a constant temperature, and the results were visually observed. The results were read by two people. The lowest concentration of the drug-free wells at the lowest drug concentration was the minimum inhibitory concentration (MIC). Each strain was tested three times.

### 3.3. Docking Studies

Docking studies of all derivatives were implemented in the program AutoDock 4.2.6 [21]. The X-ray crystallographic structure of SE (PDB ID: 2AIB) was taken from PDB (www.rcsb.org/pdb) and was invoked as a protein structure [22,23]. Beginning of docking, all the water and ligands were removed and the random hydrogen atoms were added. All the compounds were constructed using ChemDraw Ultra 14.0. Most parameters were default values except the calculation for the Lamarckian genetic algorithm (LGA). The grid box for docking size was set to 55 × 55 × 55 points in x, y and z directions. The docking experiment was implemented by AutoDock program. There were 100 multiples to independent docking runs, then each experiment was repeated 20 times, to obtain the best performance of the docking programs. Molecular dynamics of the complexes resulting from docking of compounds **13**–**17** were performed using Discovery Studio 3.5 Module Standard Dynamic Cascade (Sun Yat-sen University, Guangzhou, China). The CHARMm force field and the Moony–Rone charge were added to the composite, and then the H_2_O molecules and the NaCl molecules were added to make the NaCl concentration of the complex system 0.145, which was reached the osmotic pressure of the human body. After an initial default relaxation protocol, an MD production run was made with a time step of 2.0 ps [24].

## 4. Conclusions

In conclusion, our strategy to introduce antifungal pharmacophore allylamine into the methyl phloroglucinol and remain acyl group in the C-6 position using pseudoaspidinol as the lead compound to obtain novel phloroglucinol derivatives **4**–**17** was successful in leading to compounds, which showed inhibition of *T. mentagrophytes* and *T. rubrum*. Furthermore, the first basic efficacy analysis of Naftifine groups in the study. These compounds could be useful as lead compounds in developing novel antifungal agents.

## Figures and Tables

**Table 1:** Antifungal activities of compounds on *Trichophyton rubrum* (Tr) and *Trichophyton mentagrophytes* (Tm).

Compound	*Trichophyton rubrum* (MIC)	*Trichophyton mentagrophyte* (MIC)
**1**	41.38 μg/mL	33.63 μg/mL
**2**	19.57 μg/mL	39.82 μg/mL
**3**	160 μg/mL	160 μg/mL
**4**	160 μg/mL	160 μg/mL
**5**	160 μg/mL	160 μg/mL
**6**	62.5 μg/mL	62.5 μg/mL
**7**	39.89 μg/mL	39.72 μg/mL
**8**	160 μg/mL	160 μg/mL
**9**	78.14 μg/mL	79.53 μg/mL
**10**	160 μg/mL	160 μg/mL
**11**	36.42 μg/mL	37.46 μg/mL
**12**	37.23 μg/mL	39.54 μg/mL
**13**	39.24 μg/mL	39.17 μg/mL
**14**	160 μg/mL	160 μg/mL
**15**	5.12 μg/mL	8.13 μg/mL
**16**	160 μg/mL	160 μg/mL
**17**	3.05 μg/mL	5.13 μg/mL
Pseudoaspidinol	21.06 μg/mL	19.89 μg/mL
Naftifine	1.036 μg/mL	1.072 μg/mL

**Table 2:** Docking results of synthesized compounds.

Compounds	(−) Binding Energy ΔG (kcal/mol) 2AIB
**8**	6.23
**9**	5.65
**10**	6.02
**11**	6.18
**12**	5.98
**13**	6.00
**14**	6.44
**15**	9.93
**16**	11.27
**17**	11.80
Pseudoaspidinol	5.75
Naftifine	9.32

## Supplementary Materials

The following are available online, ^1^H-NMR, ^13^C-NMR and MS of compounds **2**–**17**.
