# Supplementary material for: Antifungal Agents: Design, Synthesis, Antifungal Activity and Molecular Docking of Phloroglucinol Derivatives
Source: Molecules. 2018 Nov 28;23(12):3116. doi: 10.3390/molecules23123116 (PMC6321598; doi:10.3390/molecules23123116)
Supplement: Supplementary file 1 [file molecules-23-03116-s001.pdf]

Title: Antifungal agents: Design, synthesis, antifungal activity and molecular docking of pseudoaspidinol derivatives

Authors: Xingxing Teng, Yuanyuan Wang, Jinhua Gu, Peiqi Shi, Zhibin Shen and Lianbao Ye

^{1}H NMR,^{13}C NMR and MS of compounds

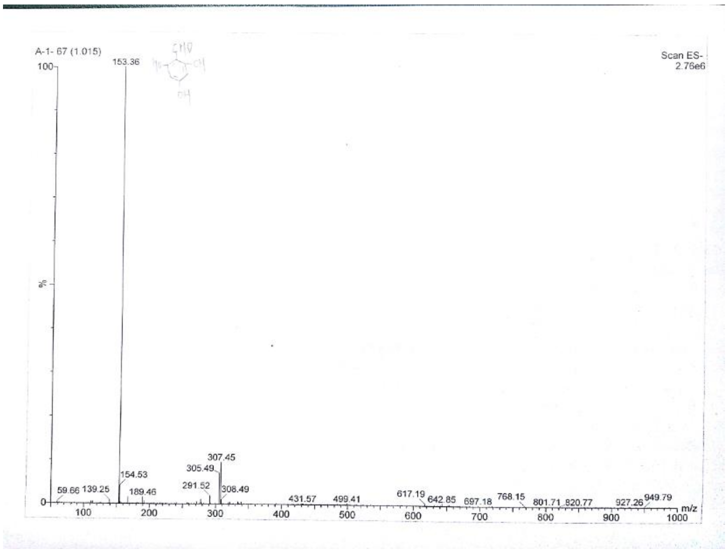

MS of compounds 2

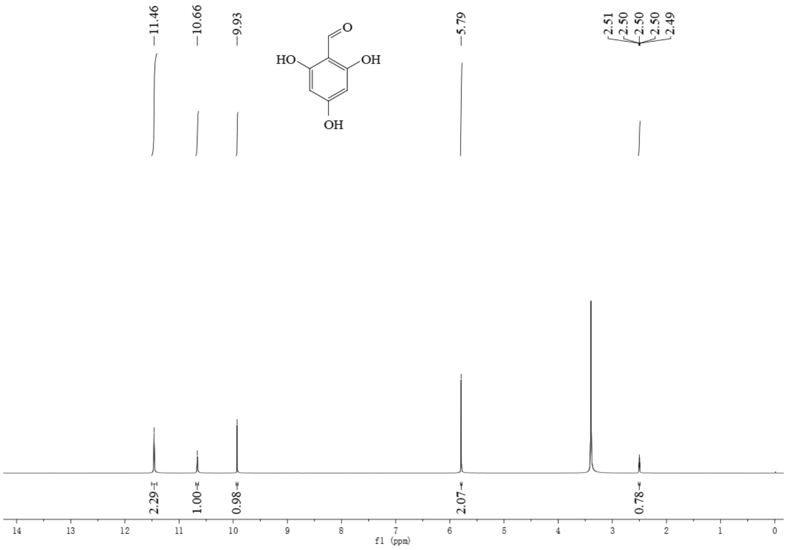

^{1}H NMR of compounds 2

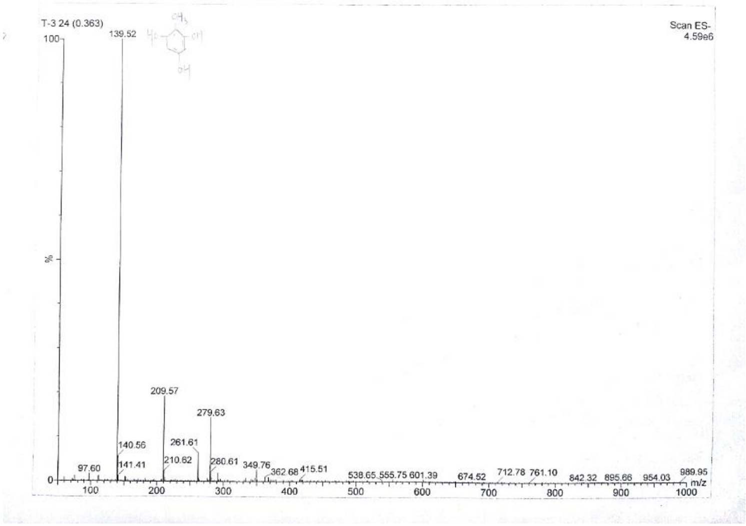

MS of compounds 3

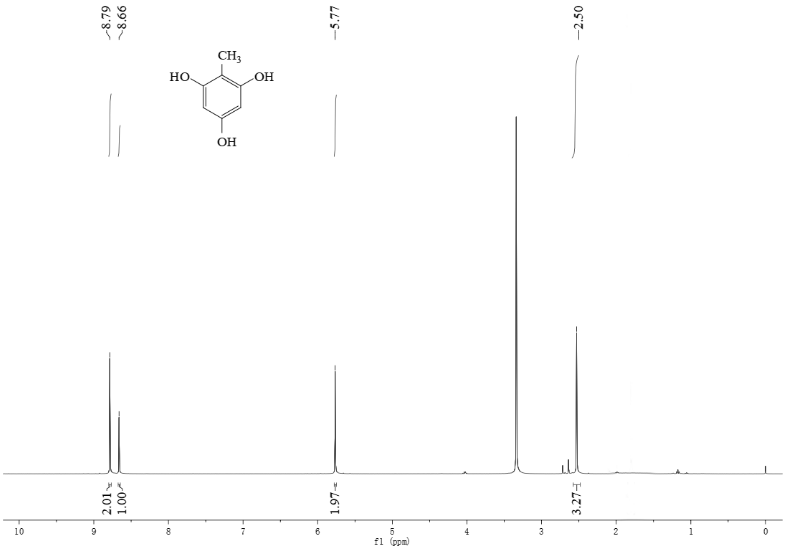

^{1}H NMR of compounds 3

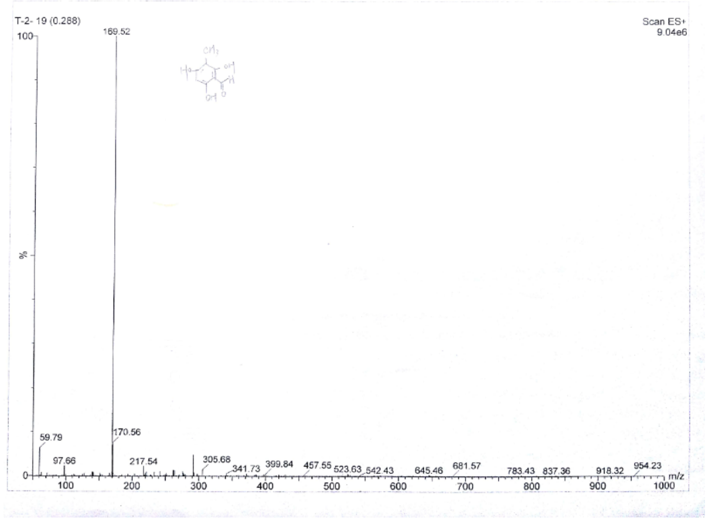

MS of compounds 4

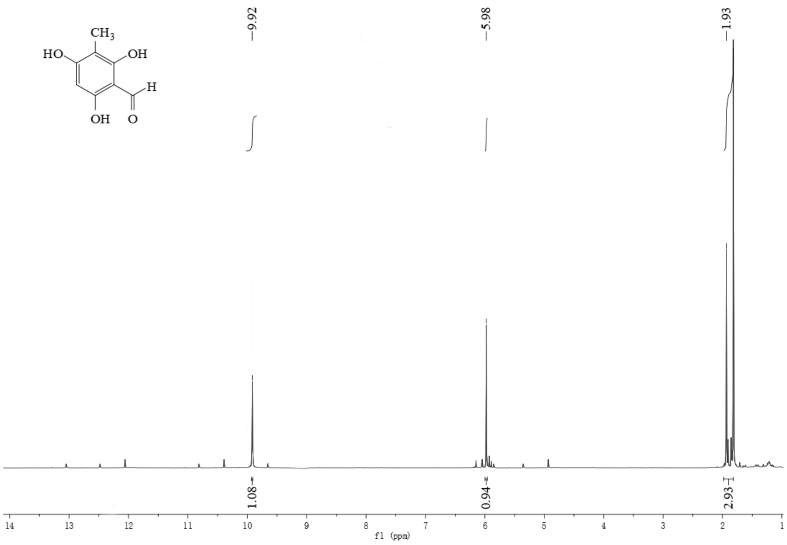

^{1}H NMR of compounds 4

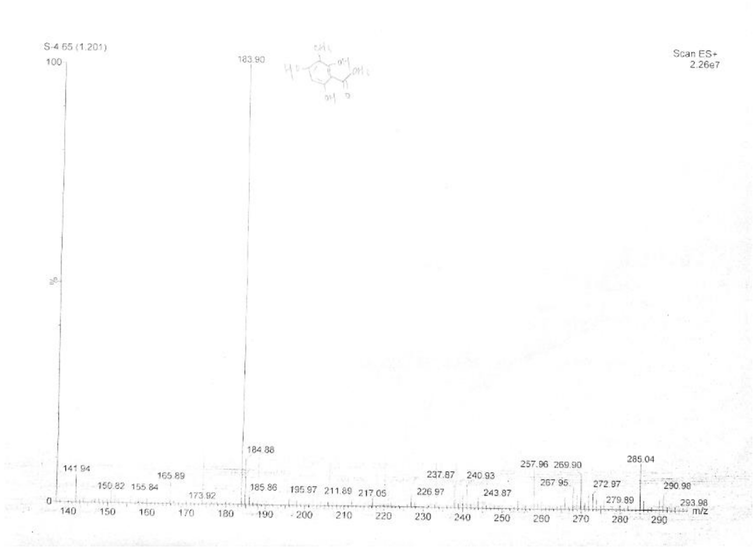

MS of compounds 5

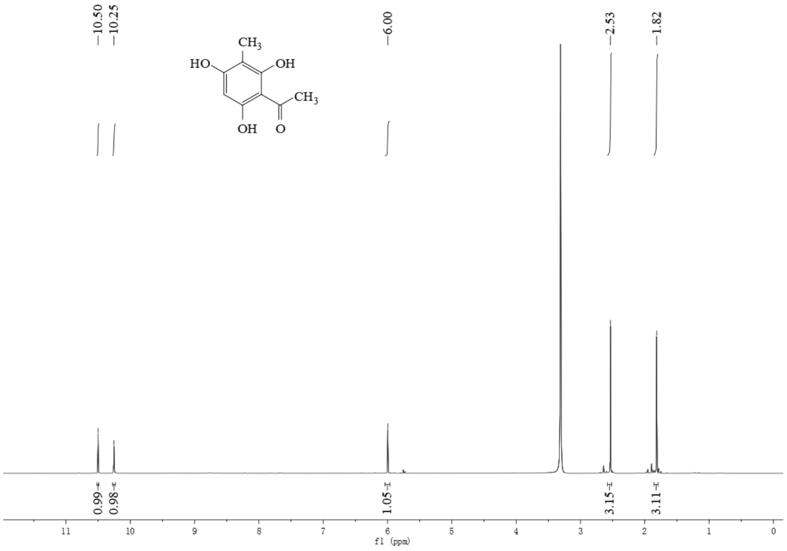

^{1}H NMR of compounds 5

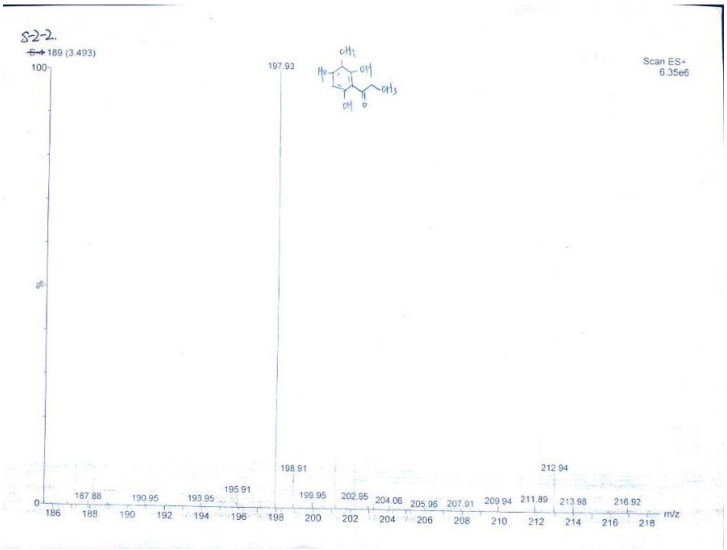

MS of compounds 6

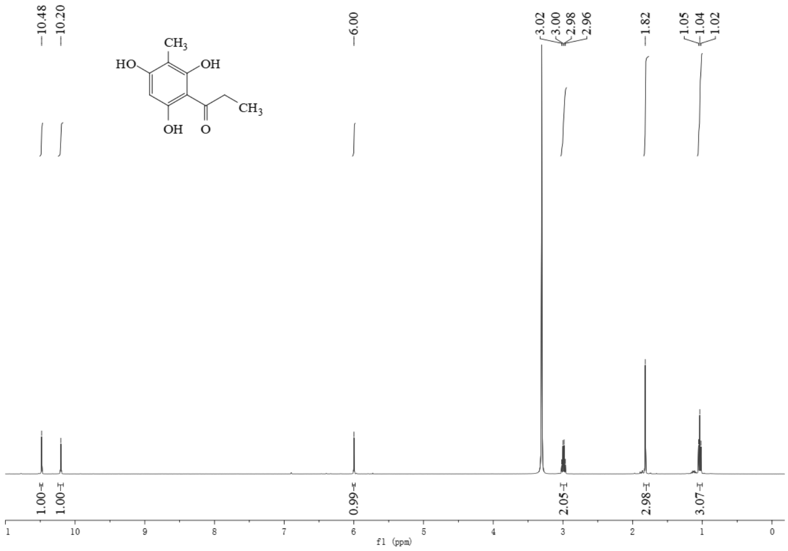

^{1}H NMR of compounds 6

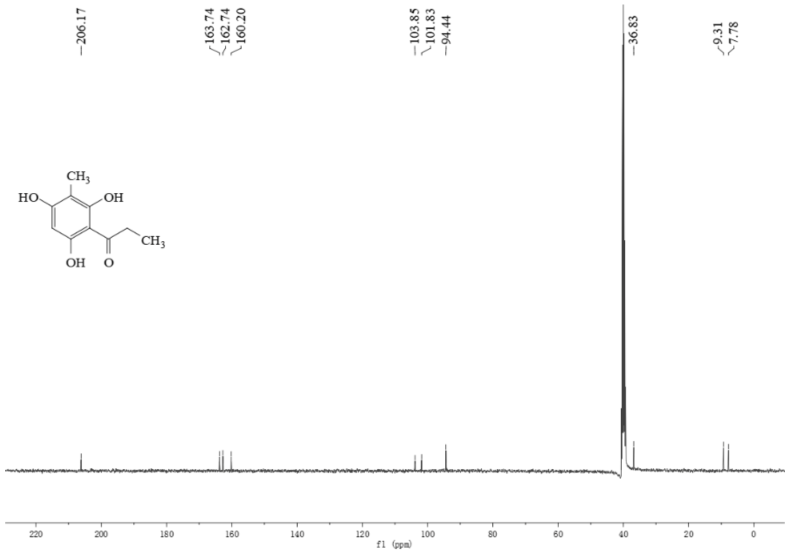

^{13}C NMR of compounds 6

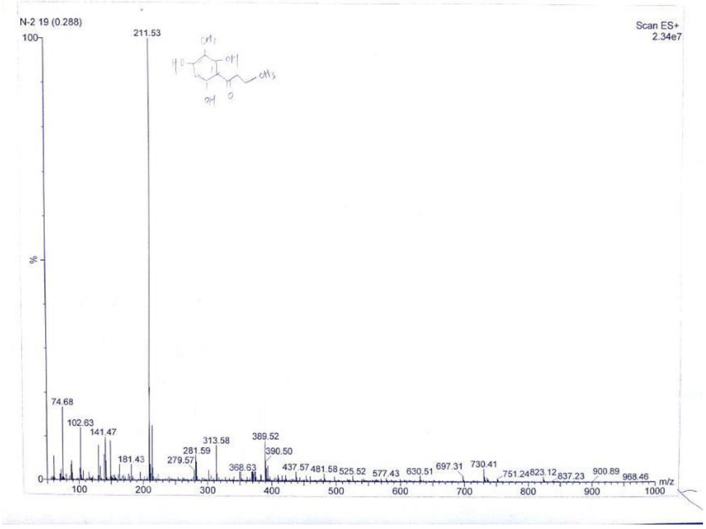

MS of compounds 7

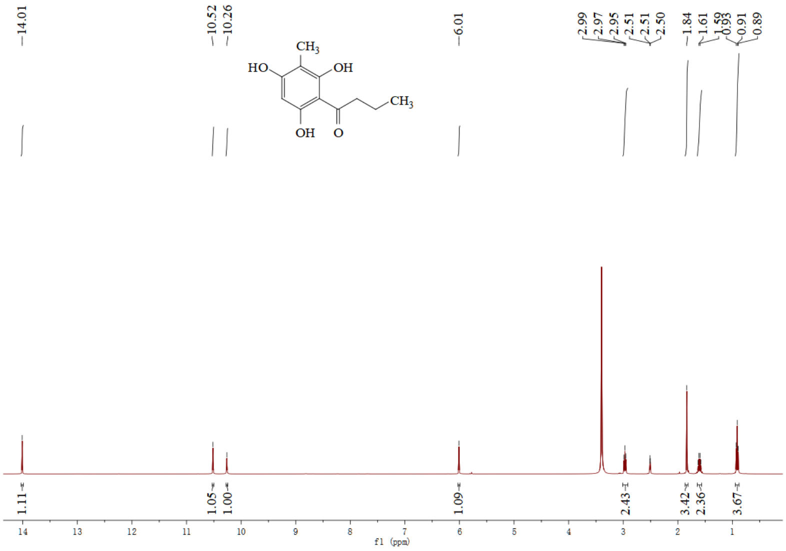

^{1}H NMR of compounds 7

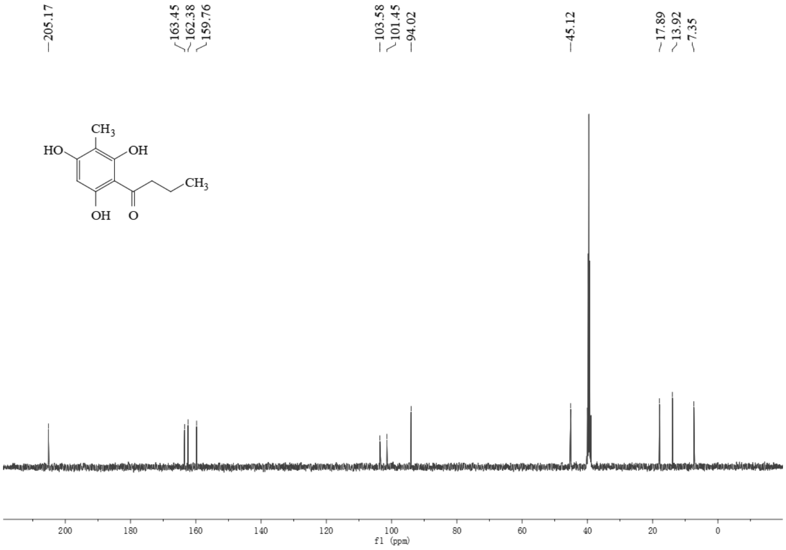

^{13}C NMR of compounds 7

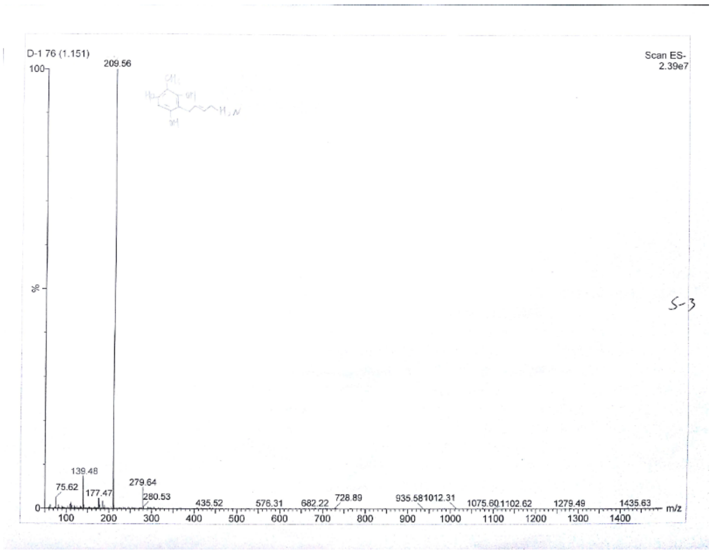

MS of compounds 8

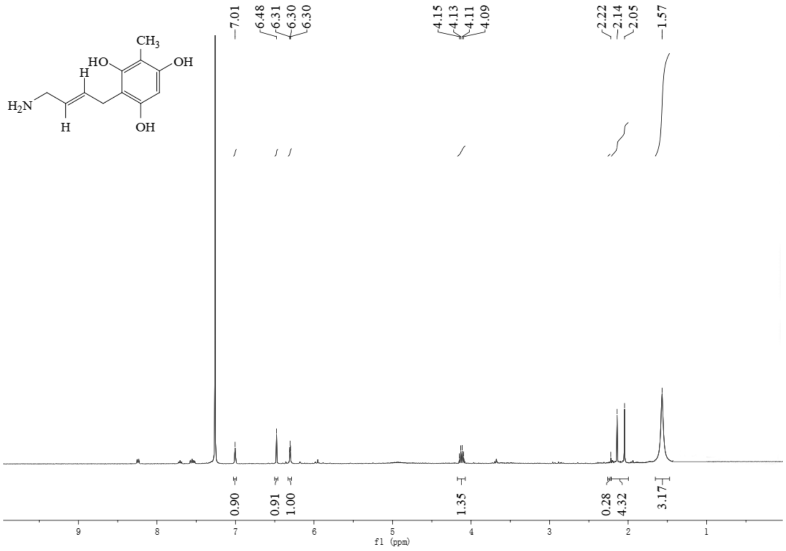

^{1}H NMR of compounds 8

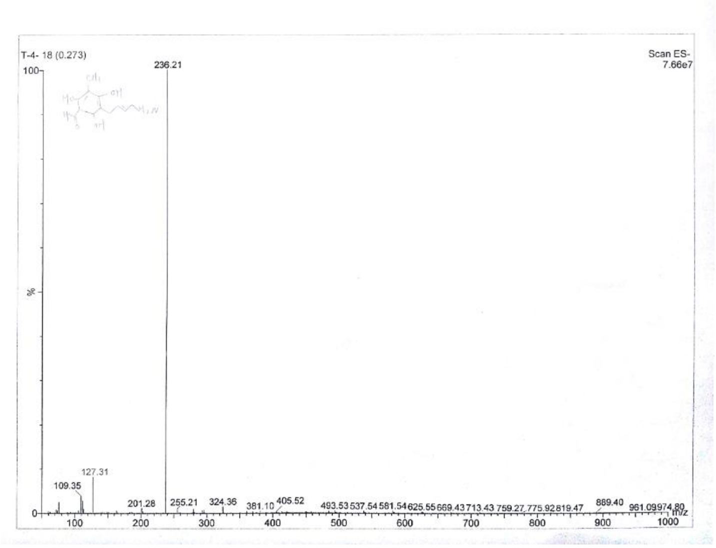

MS of compounds 9

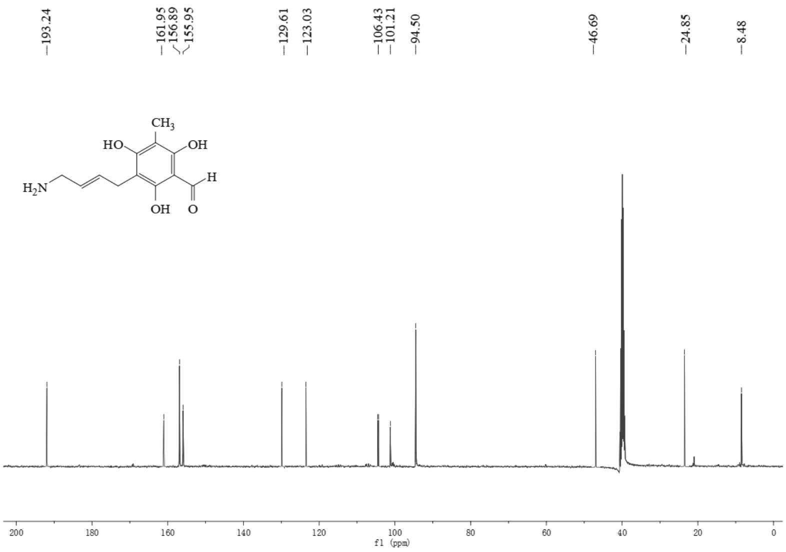

^{13}C NMR of compounds 9

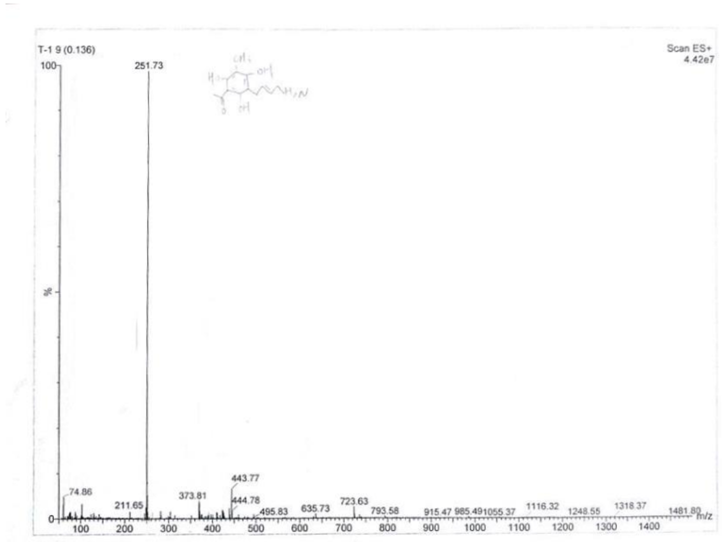

MS of compounds 10

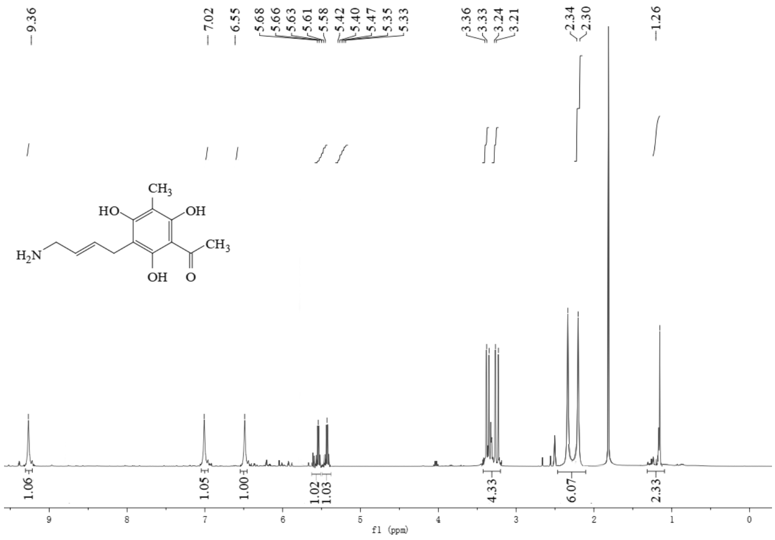

^{1}H NMR of compounds 10

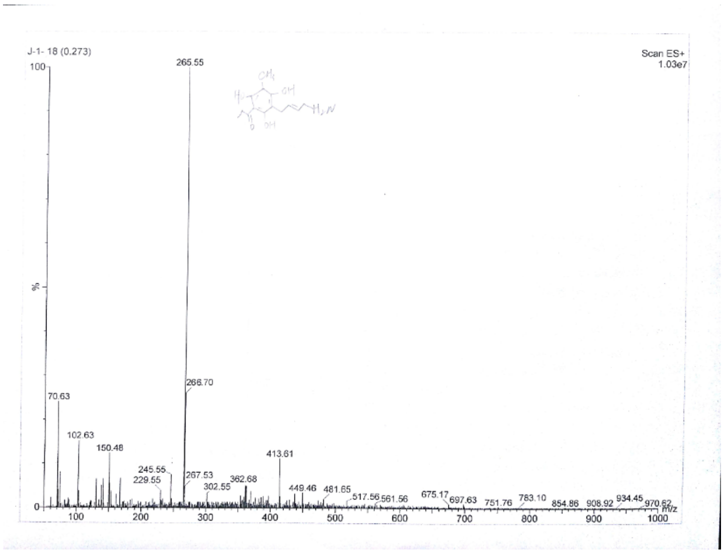

MS of compounds 11

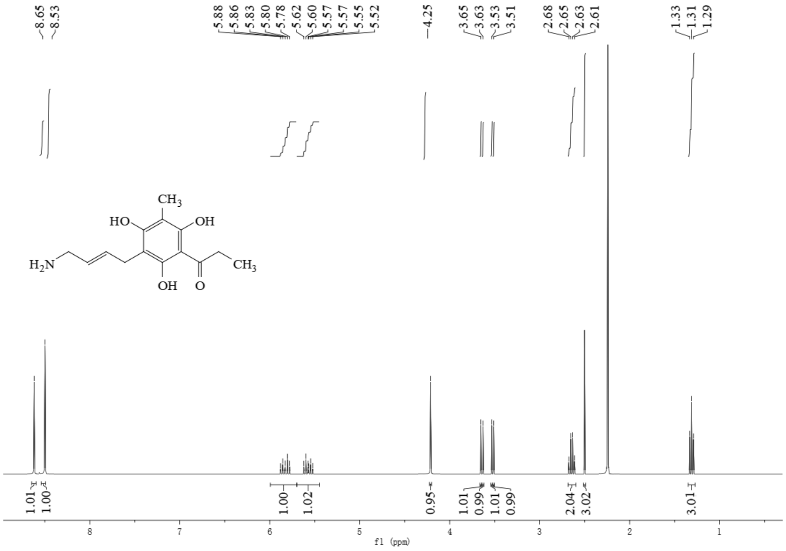

^{1}H NMR of compounds 11

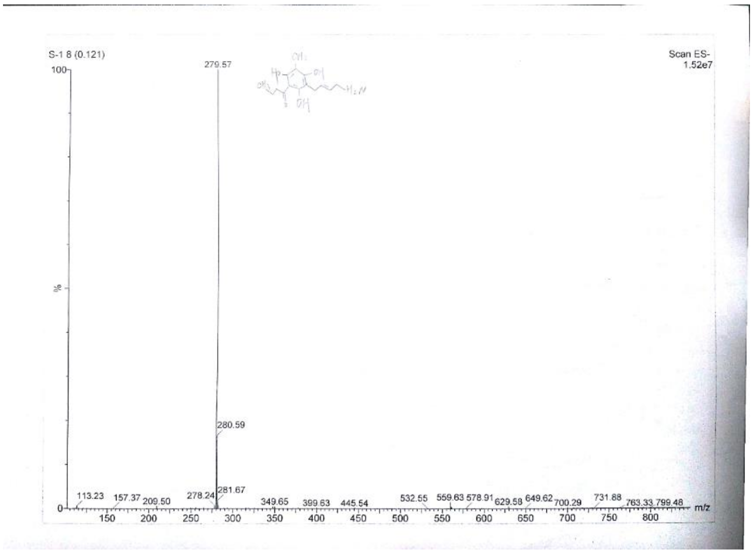

MS of compounds 12

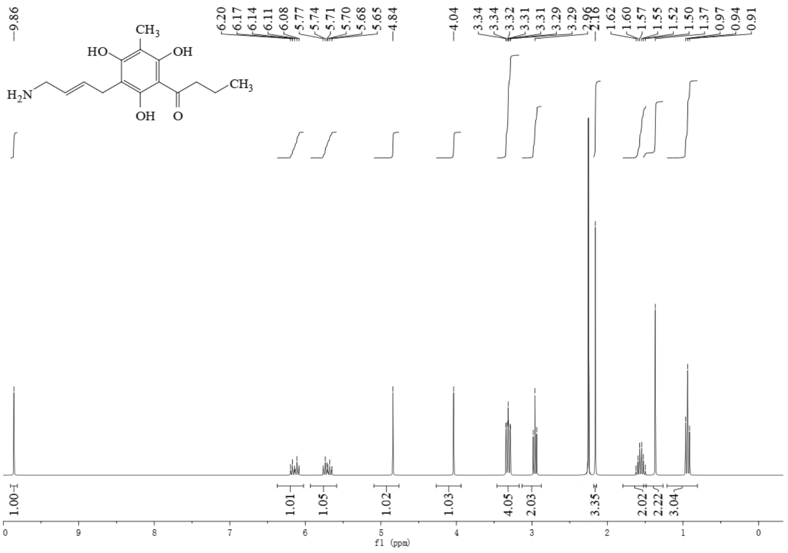

^{1}H NMR of compounds 12

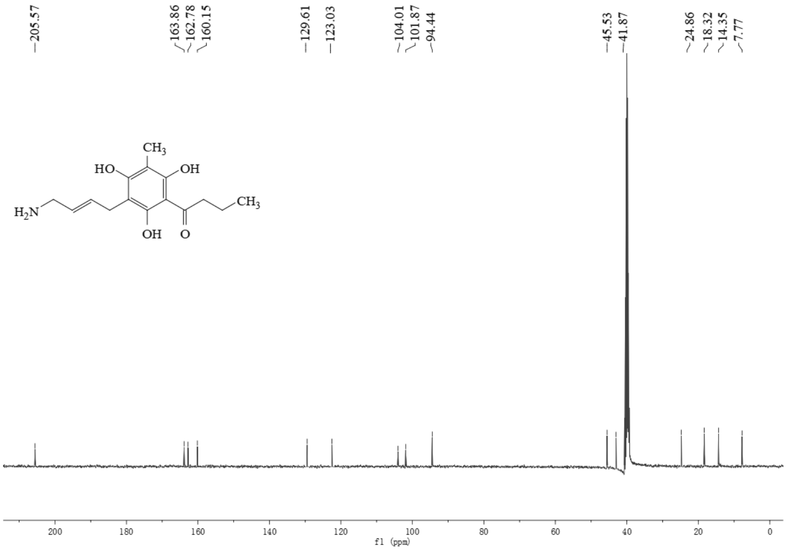

^{13}C NMR of compounds 12

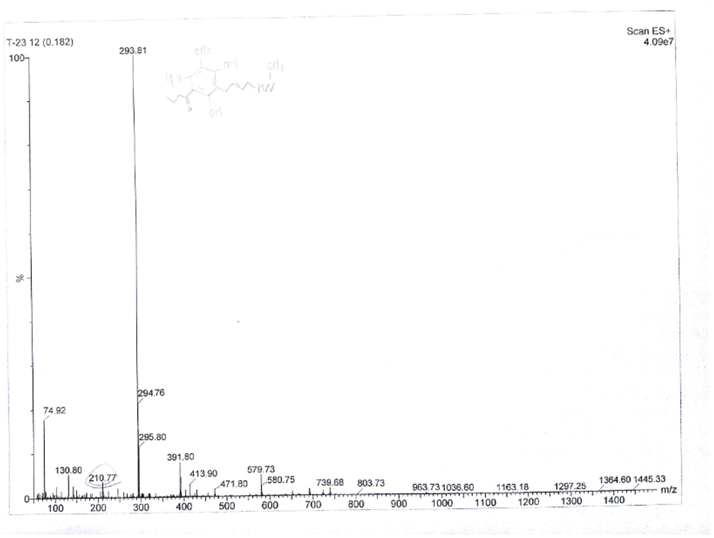

MS of compounds 13

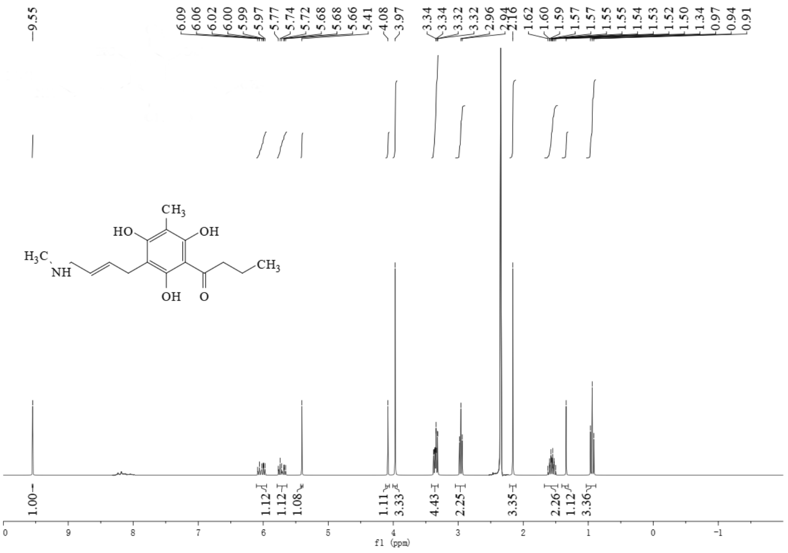

^{1}H NMR of compounds 13

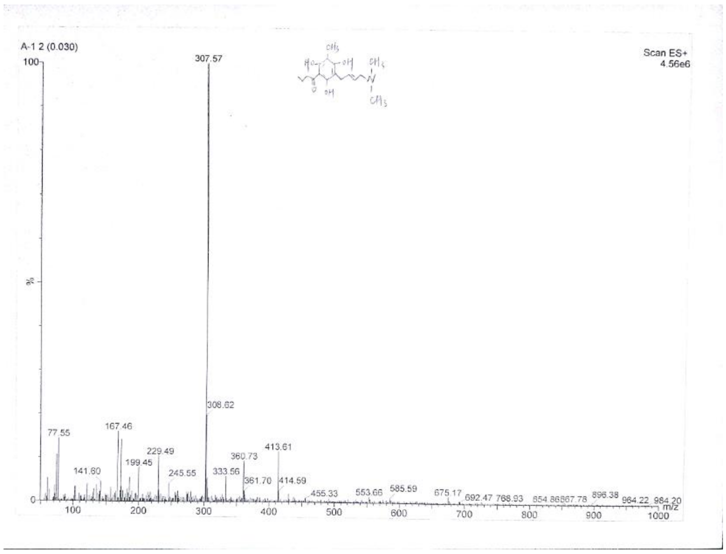

MS of compounds 14

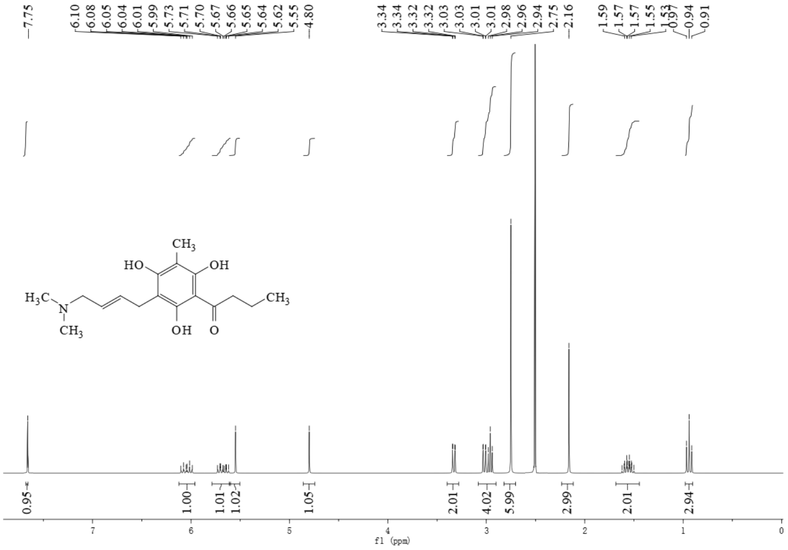

^{1}H NMR of compounds 14

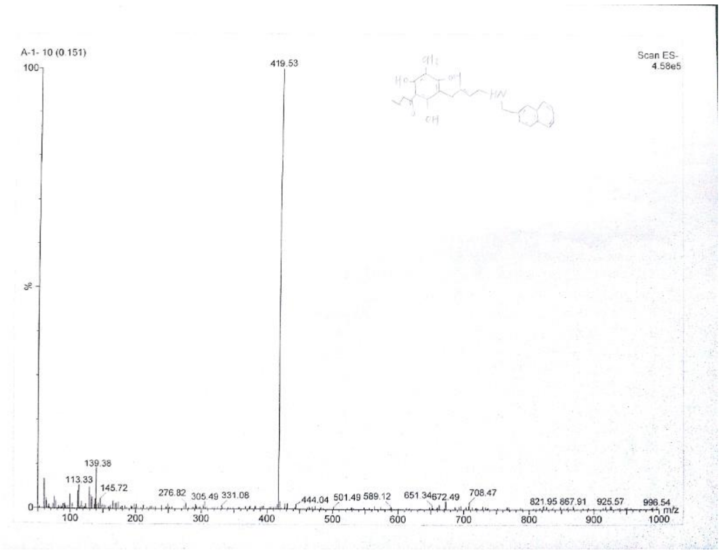

MS of compounds 15

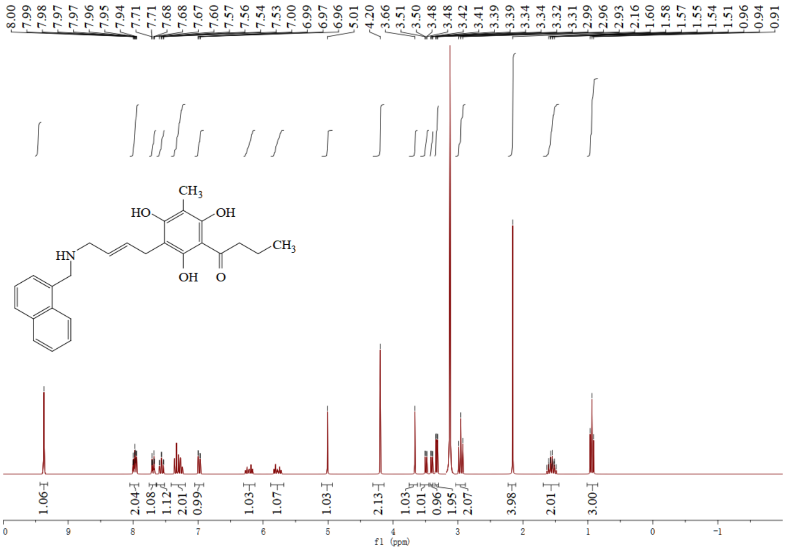

^{1}H NMR of compounds 15

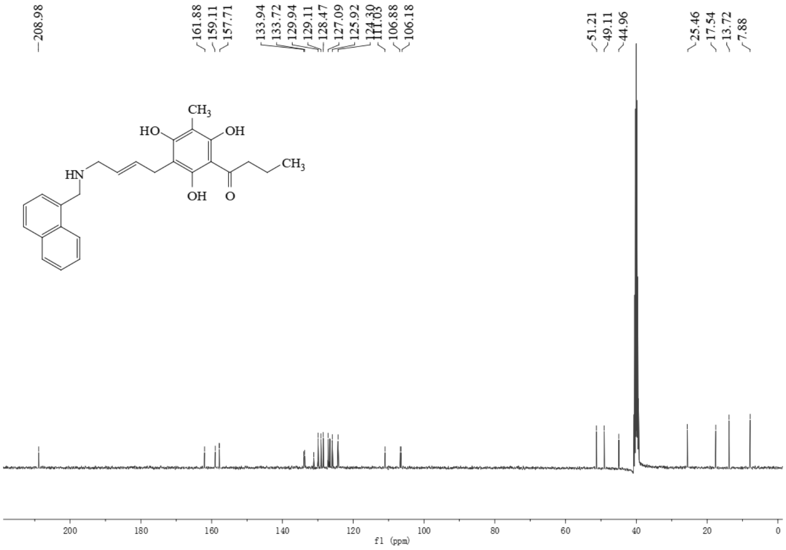

^{13}C NMR of compounds 15

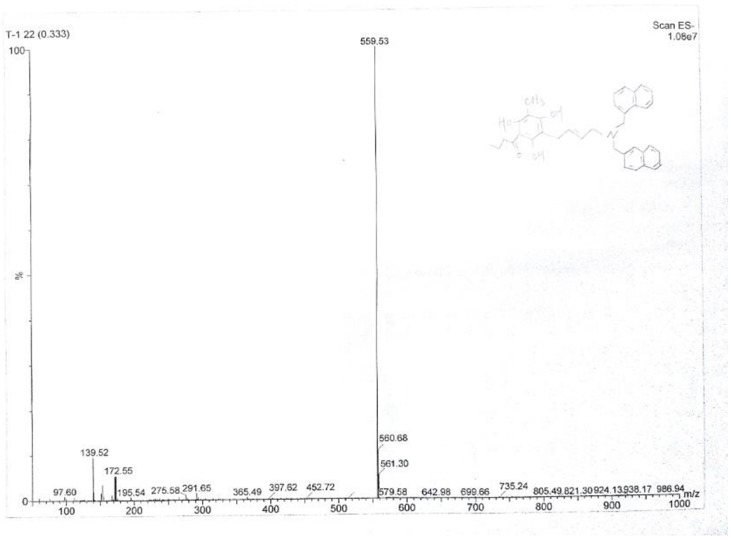

MS of compounds16

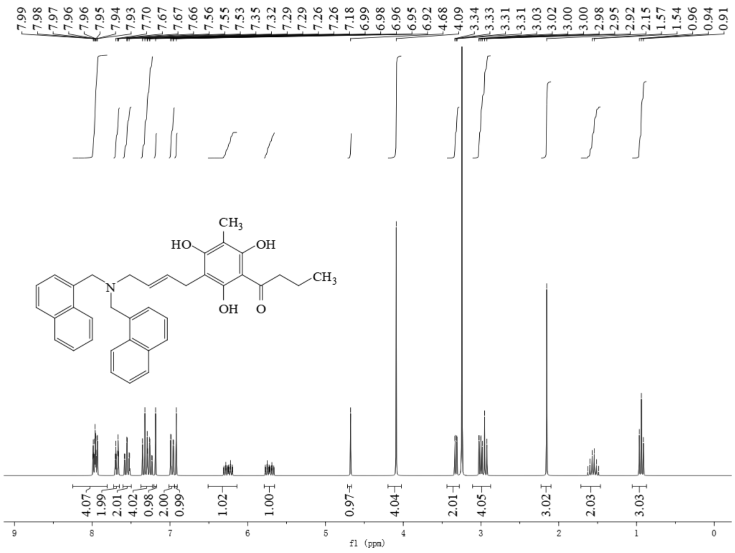

^{1}H NMR of compounds 16

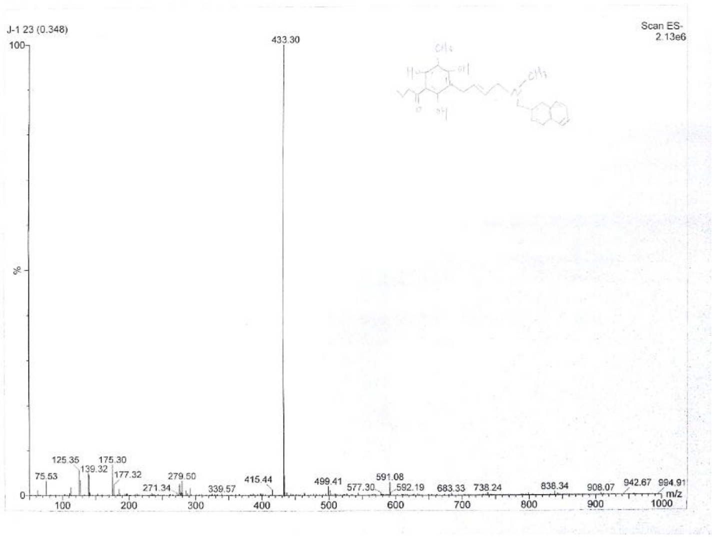

MS of compounds 17

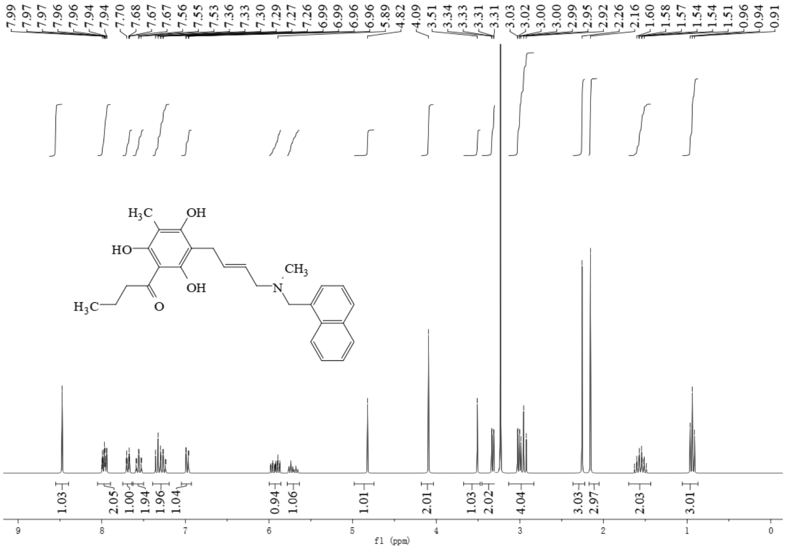

^{1}H NMR of compounds 17

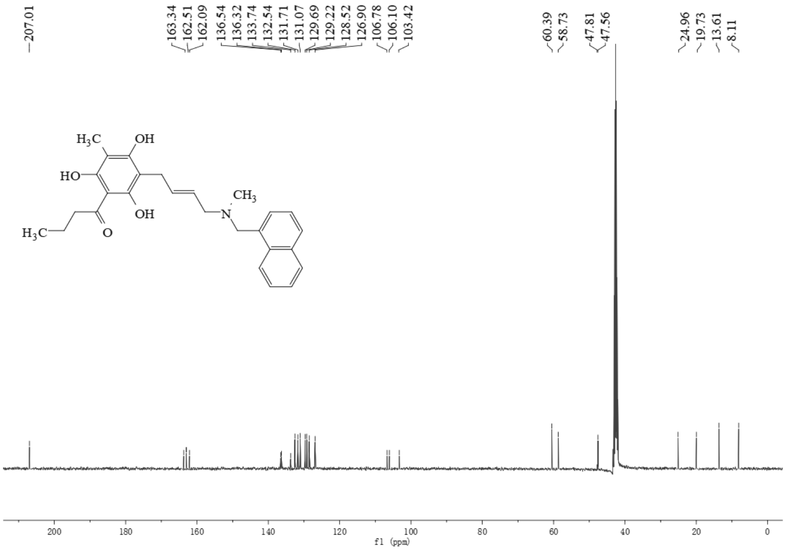

^{13}C NMR of compounds 17
